# Color-Blindness a Brain Affection

**Published:** 1888-01

**Authors:** 


THE NEW YORK CANCER HOSPITAL.—The New York Cancer Hospital, which was formally opened this week for the reception of patients, is the only one of its kind in this country, and bids fair, with the means to the end of treating the most dreaded of all diseases, to mark an era in the history of public institutions in this city. Desirably situated, handsomely built, and thoroughly equipped as it is, it gives a reasonable promise of great usefulness. With the objects, not only of affording relief for a large class of sufferers, but of stimulating research and perfecting skill, it certainly lays claim to the heartiest good wishes of every lover of humanity, and the best encouragement to every scientific student of our noble art. It is not fair to assume that because cancer is now, as a rule, incurable, that we may not yet find some remedy as efficacious for its successful treatment as is bark for intermittents and mercury for syphilis. At least we should be tireless in our endeavors to make such a realization possible. The Cancer Hospital, dedicated to such high purposes, modestly and earnestly appeals to the profession of this country for aid and encouragement. * * * —*Medical Record.*

---

COLOR-BLINDNESS A BRAIN AFFECTION.—According to our scientific cotemporary, *Nature*, Professor W. Ramsay urges, in a recent issue of the Proceedings of the Bristol Naturalists' Society, that the particular defect giving rise to color-blindness lies, not in the eye itself, but in the brain. Certain persons, he points out, are incapable of judging which of two musical tones is the higher, even when they are more than an octave apart. Yet as such persons hear either tone perfectly the defect is not one of deafness. He accordingly argues that in such persons the brain is at fault, and thence proceeds to the assumption that it may be equally true that the inability to perceive certain colors is not due to a defect in the instrument of sight by the eye, but to the power of interpreting the impressions conveyed to the brain by the optic nerve. If this is the case the problem is no longer a physical one, it falls among those with which the mental physiologist has to deal.—*Medical Press and Circular.*

